# Awareness of Obesity and Weight Loss Management Among Adults in the Asir Region, Saudi Arabia

**DOI:** 10.7759/cureus.75066

**Published:** 2024-12-03

**Authors:** Abdullah M Al-Shahrani, Saif M Alqahtani, Mada A Alghamdi, Hadi Albalhsn, Lama A Almalhan, Malik M Alamri, Abdullah S Alshahrani, Abdulrahim S Alamri, Alwaleed M Alqahtani, Saeed M Alzhrani, Manar M Alqahtani, Elhadi Miskeen

**Affiliations:** 1 Family Medicine, College of Medicine, University of Bisha, Bisha, SAU; 2 Medicine, College of Medicine, University of Bisha, Bisha, SAU; 3 Surgery, College of Medicine, University of Bisha, Bisha, SAU; 4 College of Medicine, King Khalid University, Abha, SAU; 5 Obstetrics and Gynecology, College of Medicine, University of Bisha, Bisha, SAU

**Keywords:** dietary management, management of obesity, obesity, physical activity, weight loss

## Abstract

Obesity, a chronic disease marked by excessive fat accumulation and a body mass index (BMI) of 30 kg/m² or more, has become a major global health issue, affecting many adults worldwide and particularly prevalent in developed nations and Saudi Arabia. The condition can be caused by genetic, metabolic, and lifestyle factors. Understanding its awareness is imperative in designing effective health interventions. A cross-sectional study was done to assess awareness of obesity and weight loss management among adults in the Asir region of Saudi Arabia. A total of 638 participants were included in this study, a self-administered electronic questionnaire was distributed to 638 respondents, and data were analyzed using SPSS version 27. Our study revealed low practical adherence with 83.5% (533 participants) not following any specific diet and only 29.8% (190 participants) engaging in regular physical activity. Sleep and hydration patterns varied, with 51.9% (331 participants) of participants sleeping fewer than seven hours and 61.9% (395 participants) not meeting recommended water intake. While 66.1% (422 participants) acknowledged that medical conditions such as metabolic syndrome can contribute to obesity, a significant proportion lacked knowledge about the impact of medications and stress. Dietary habits showed that 24.5% (156 participants) practiced intermittent fasting, yet only 1.7% (11 participants) had undergone weight loss surgery. Gender, age, body weight, BMI, and self-perception of weight were significantly associated with knowledge levels, with females, younger participants, and those with normal BMI exhibiting higher awareness. There was reasonable awareness of obesity and weight loss management. However, practical adherence to healthy behaviors, such as regular physical activity and dietary management, remains low. Most participants recognize lifestyle-related factors contributing to obesity and favor non-surgical, non-medication-based weight management. Healthcare professionals should organize seminars and educational programs to enhance public understanding of obesity and weight management in Saudi Arabia.

## Introduction

Obesity, a chronic, relapsing disease, is a major health issue that has increasingly become more prevalent in recent decades [1]. The disease has several possible causes, including but not limited to genetic, metabolic, and environmental factors [2]. The condition is marked by an abnormal accumulation of fat leading to an excessive increase in a person’s weight, attaining a body mass index (BMI) of 30 kg/m^2^ or more [3]. According to the World Health Organization, about 890 million people were obese globally in 2022, with 16% of adults aged 18 and above living with obesity [3]. The prevalence of obesity has been particularly increasing in the developed world, with about 41.9% of adults in the United States being affected by obesity, while about 10.3% of adults aged 20 and above are affected by severe obesity [4]. In Europe, about 60% of adults are either overweight or obese [5]. 

In Saudi Arabia, obesity has also become a huge challenge, with about 23.7% of people aged 15 and above being diagnosed with obesity [6]. According to the WHO, Saudi Arabia has the second highest prevalence of obesity in the Gulf region. Poor dietary habits and technology-inspired sedentary lifestyles are the main reasons for the recent upsurge in obesity [7]. Given the level of these lifestyle changes, coupled with other factors such as genetic and environmental factors, the challenge of obesity may continue to be even more of a public health concern in the future. Obesity comes with a significant burden because not only does it lead to increased medical costs but also causes decreased productivity among patients [8]. In addition, obesity is associated with other healthcare challenges, such as diabetes, non-alcoholic fatty liver disease (NAFLD), cancer, chronic kidney disease, and many others [9]. Obesity and all the other related conditions significantly reduce patients’ quality of life and life expectancy, given that obesity is linked to many premature deaths globally. 

Given the great public health threat posed by obesity, it is important that all people have adequate awareness of the condition and have sufficient information regarding avoiding it and managing weight. Saudi Arabia, being one of the most developed countries in the Gulf region, has been proven to have a major overweight and obesity challenge that poses a significant threat to the future sustainability of the country. In this regard, this study seeks to explore the awareness of obesity and weight loss management among adults in the Asir region of Saudi Arabia. 

## Materials and methods

Study design 

A community-based cross-sectional study was designed to assess awareness and perceptions toward obesity and weight loss management among adults in the Asir region, Saudi Arabia. The research was conducted over seven months between the period of April 2024 to October 2024. 

Inclusion and exclusion criteria

The study targeted adult residents of the Asir region, Saudi Arabia, aged 18 and above, inclusive of both genders. Inclusion criteria required participants to be willing to participate and capable of completing an online survey independently. Exclusion criteria included individuals under the age of 18, pregnant women, individuals who participated in the pilot study, and those who declined participation.

Sample size and sampling technique 

The study employed a non-probability convenience sampling method in recruiting participants. The sample size was determined using the Cochrane sample size formulae with a 95% confidence level and a 5% margin of error, resulting in a minimum acceptable sample size of 384. However, to improve the reliability, a higher sample of 638 was used.

Data collection tools and methods 

Data were collected using a self-administered electronic questionnaire distributed via Google Forms (Google LLC, Mountain View, California, United States). The questionnaire, modified to suit the study objectives, was reviewed by two research faculty experts from the University of Bisha. A pilot study involving 30 participants was conducted to ensure the questionnaire’s clarity and relevance. These participants were excluded from the main study. The pilot yielded a Cronbach's alpha of 0.841, indicating high internal consistency. The questionnaire was distributed via WhatsApp groups and promoted by local social influencers to encourage participation. The survey collected sociodemographic information and assessed participants’ awareness regarding obesity, weight loss management, and history of weight loss surgery. All questions and consent forms were translated into Arabic to facilitate understanding among participants and also back translation was done to English.

Data analysis 

Microsoft Excel spreadsheets (Microsoft Corporation, USA) were used to do all the data cleaning. Then, data were transferred to IBM SPSS Statistics for Windows, Version 27.0 (released 2020, IBM Corp., Armonk, NY) where the analysis was conducted. As the data were categorical, it was presented in tabular form, displaying counts and frequencies. Appropriate statistical tests like the chi-square test were applied to determine statistical significance set at p < 0.05.

Ethical considerations 

Ethical approval for the study was obtained from the University of Bisha Institutional Review Board (IRB) (approval no. UB-RELOC H-06-BH-087/(1805.24)). No study activities commenced before securing IRB approval. All participants were informed about the study's objectives, and individual informed consent was obtained prior to survey completion. Participants were assured of their right to withdraw from the study at any point, with confidentiality and anonymity maintained throughout. Respondents’ data were protected, and their right to refuse or withdraw from the study was respected. 

## Results

Table 1 provides an overview of the sociodemographic and self-reported characteristics of the 638 participants in this study. Females represented the majority of the sample at 69.6%, with males comprising 30.4%. The largest age group was 18-20 years (38.6%). In terms of the BMI, 47.8% of the participants had a normal BMI (18.5-25), while a smaller proportion, 2.2%, had a BMI over 40. Regarding weight perception, 48.8% of the participants considered their weight to be normal, 39% felt they were overweight, and 5.8% reported feeling very overweight. 

**Table 1:** Sociodemographic characteristics of the respondents

Variable	Category	Count	Percentage (%)
Gender	Female	444	69.6
	Male	194	30.4
Age	18-20	246	38.6
	21-30	232	36.4
	31-40	59	9.2
	41-50	64	10
	Above 50	37	5.8
Weight (kg)	Above 109	15	2.4
	50-69	290	45.5
	70-89	161	25.2
	90-109	50	7.8
	Less than 50	122	19.1
Height (cm)	Above 179	24	3.8
	150-159	243	38.1
	160-169	233	36.5
	170-179	112	17.6
	Less than 150	26	4.1
Body mass index (kg/m²)	Above 40	14	2.2
	18.5-25	305	47.8
	26-30	119	18.7
	31-35	50	7.8
	36-40	40	6.3
	I don’t know	22	3.4
	Less than 18.5	88	13.8
Personal perception about weight	I am overweight	249	39
	I feel very fat	37	5.8
	Less than normal	41	6.4
	My weight is normal	311	48.8
How often do you check your weight	Every day	38	6
	Every week	122	19.1
	I don’t check my weight	80	12.5
	Infrequently	291	45.6
	Per Month	107	16.8

Table 2 shows that an overwhelming majority (83.5%) reported not following any specific diet, while only 16.5% adhered to a dietary regimen. Regular physical activity was also low, with just 29.8% engaging in regular exercise. Among those who exercised, only 4.7% exercised daily with the majority nearly half of them (47%) not exercising at all. Sleep patterns varied, with slightly more than of the respondents (51.9%) sleeping between three and six hours per day and 46.1% achieving seven hours or more. Regarding hydration, 61.9% of the participants did not meet the recommended daily water intake, with only 38.1% meeting or exceeding the recommended levels. Finally, in terms of daily activity levels, 44.8% described their lifestyle as lightly active, followed by 30.3% who were moderately active, while a smaller group was sedentary (19.4%) or very active (5.5%). 

**Table 2:** Participants’ lifestyle that affect their health

Research item	Variable	Count	Percentage (%)
Do you follow any diet?	No	533	83.5
	Yes	105	16.5
Do you practice any kind of sport regularly?	No	448	70.2
	Yes	190	29.8
How many days do you exercise during the week?	3 to 4 days a week	95	14.9
	5 to 6 days a week	50	7.8
	Everyday	30	4.7
	I don’t exercise	300	47
	1 to 2 days a week	163	25.6
How many hours do you sleep per day?	1 to 2 hours	13	2.0
	7 hours or more	294	46.1
	From 3 to 6 hours	331	51.9
Did you drink the recommended water intake per day: for men (3.7 Liters) and for women (2.7 Liters)	No	395	61.9
	Yes	243	38.1
Your activity level in a day	Sedentary	124	19.4
	Light active	286	44.8
	Moderately active	193	30.3
	Very active	35	5.5

Table 3 depicts a notable lack of knowledge among the respondents regarding certain medications, such as antidepressants, nearly half (49.1%) believe that can lead to obesity, while 8.6% disagreed, and 42.3% were unsure. A majority (66.1%) agreed that medical conditions like metabolic syndrome, polycystic ovary syndrome, and hypothyroidism can result in weight gain, with only 4.4% disagreeing and 29.5% expressing uncertainty. Regarding the impact of stress, 45% of the participants felt that high stress levels could contribute to obesity, while 21% disagreed and 34% were unsure. Similarly, 46.2% believed that inadequate quality sleep could lead to obesity, whereas 18.8% disagreed, and 35% were uncertain. 

**Table 3:** Participants’ level of knowledge toward obesity and weight loss management The correct answers for each question are highlighted in bold to indicate the most accurate responses based on current evidence and guidelines regarding obesity and weight loss management.

Variable	True	False	Not Sure
Medications like antidepressants can cause obesity.	313 (49.1%)	55 (8.6%)	270 (42.3%)
Some conditions, such as metabolic syndrome, polycystic ovary syndrome, and hypothyroidism, cause people to gain weight.	422 (66.1%)	28 (4.4%)	188 (29.5%)
High amounts of stress can cause obesity.	287 (45%)	134 (21%)	217 (34%)
Not getting enough good-quality sleep can cause obesity.	295 (46.2%)	120 (18.8%)	223 (35%)

Table 4 shows that a small fraction of participants followed popular diets, with 1.6% following the Dukan diet and 1.4% following the Atkins diet. A larger group, 24.5%, practiced intermittent fasting, while 8.2% reported following diets other than those specifically listed. Similarly, 8.2% of the participants used herbal medicines as part of their weight loss efforts. By contrast, weight loss surgery was rare, with only 1.7% of the participants having undergone such a procedure. 

**Table 4:** Diet plans tried by the participants

Research item	Yes	No
Do you follow the Dukan diet?	10 (1.6%)	628 (98.4%)
Do you follow the Atkins diet?	9 (1.4%)	629 (98.6%)
Do you follow intermittent fasting diet?	156 (24.5%)	482 (75.5%)
Do you follow any type of diet other than those mentioned above?	52 (8.2%)	586 (91.8%)
Have you used any herbal medicines to lose weight?	52 (8.2%)	586 (91.8%)
Have you ever undergone weight loss surgery?	11 (1.7%)	627 (98.3%)

As per Table 5, the most common choices were avoidance diets (32.7%) and calorie-focused diets (21.2%), with smaller portions opting for low carbohydrate diets (19.2%) and medically recommended diets (7.7%). Regarding herbal and natural remedies for weight loss, the most frequently used were herbal teas and natural supplements like green tea and apple cider vinegar (32.7%), while others combined herbs such as ginseng, psyllium, and ginger (25%). The majority of participants (92.9%) reported not using any medication for weight loss. Among those who used medications, the most commonly used were Saxenda (1.9%), Ozempic (1.7%), Monjaro (1.6%), and other unspecified medications (1.6%). Victoza was the least used medication, with only (0.3%) of participants reporting its use.

**Table 5:** Participants’ assessment toward obesity and weight loss management Data has been presented has numbers (n) and proportion (%).

Variable	Category	Count	Percentage (%)
Any other diet type other than Dukan, Atkins, and intermittent fasting (n=52)	Low-carbohydrate diet	10	19.2
	Calorie diet	11	21.2
	Avoidance diet	17	32.7
	Medically recommended diet	4	7.7
	Others	10	19.2
Any other herbal medicine of losing weight (n=52)	Herbal remedies and natural supplements such as green tea, cumin, apple cider vinegar, etc.	17	32.7
	Dietary supplements like saxenda injection and chlorophyll	6	11.5
	Common beverages with claimed benefits like coffee peel	5	9.6
	Combination of herbs such as animal cut/ginseng/psyllium, cumin and ginger, etc.	13	25
	Others	11	21.2
Do you use any of the following medications to lose weight? (n=638)	I do not use any medication to lose weight.	593	92.9
	Monjaro	10	1.6
	Ozempic	11	1.7
	Saxenda	12	1.9
	Victoza	2	0.3
	Use other medications	10	1.6

Table 6 shows that gender differences were evident, with females showing higher levels of good knowledge (73.5%) compared to males (26.5%), with a significant p-value of 0.003. Age was also significantly associated with knowledge levels (p < 0.001), where participants aged 21-30 had the highest proportion of good knowledge (43.9%), while younger participants (18-20) were more likely to have poor knowledge (50.4%). Weight showed a significant relationship (p = 0.011), with individuals in the 50-69 kg range having a higher prevalence of good knowledge (46.4%). BMI also correlated with knowledge levels (p = 0.008); participants with a normal BMI (18.5-25) had higher knowledge levels (48.3%), while those uncertain of their BMI or in higher ranges showed lower knowledge. Self-perceptions of weight were similarly associated, as those identifying as overweight were more likely to report good knowledge (44.7%, p < 0.001) compared to those who perceived their weight as normal (43.4%). No significant associations were found for height, indicating it had minimal impact on knowledge levels. 

**Table 6:** Association between social demographic variables and participants’ total knowledge on obesity and weight loss management A chi-square test was used to determine statistical significance. A p-value < 0.05 is considered significant. The asterisk (*) indicates statistically significant associations with a p-value < 0.05. Knowledge levels were assessed based on responses to a structured questionnaire about obesity and weight loss management. Poor knowledge refers to participants scoring below 50% on the knowledge scale, while good knowledge represents scores of 50% or above.

Variable	Category	Knowledge levels		p-value
		Poor	Good	
Gender	Female	141 (62.4%)	303 (73.5%)	0.003
	Male	85 (37.6%)	109 (26.5%)	
Age	18-20	114 (50.4%)	132 (32.0%)	<0.001*
	21-30	51 (22.6%)	181 (43.9%)	
	31-40	18 (8.0%)	41 (10.0%)	
	41-50	25 (11.1%)	39 (9.5%)	
	More than 50	18 (8.0%)	19 (4.6%)	
Weight (kg)	Above 109	8 (3.5%)	7 (1.7%)	0.011
	50-69	99 (43.8%)	191 (46.4%)	
	70-89	45 (19.9%)	116 (28.2%)	
	90-109	17 (7.5%)	33 (8.0%)	
	Less than 50	57 (25.2%	65 (15.8%)	
Height (cm)	Above 179	11 (4.9%)	13 (3.2%)	0.272
	150-159	77 (34.1%)	166 (40.3%)	
	160-169	85 (37.6%)	148 (35.9%)	
	170-179	40 (17.7%)	72 (17.5%)	
	Less than 150	13 (5.8%)	13 (3.2%)	
Body mass index (kg/m²)	Above 40	4 (1.8%	10 (2.4%)	0.008
	18.5-25	106 (46.9%)	199 (48.3%)	
	26-30	32 (14.2%)	87 (21.1%)	
	31-35	16 (7.1%)	34 (8.3%)	
	36-40	21 (9.3%)	19 (4.6%)	
	I don’t know	14 (6.2%)	8 (1.9%)	
	Less than 18.5	33 (14.6%)	55 (13.3%)	
Personal opinion about your weight	I am overweight.	65 (28.8%	184 (44.7%)	<0.001*
	I feel very fat.	8 (3.5%)	29 (7.0%)	
	Less than normal	21 (9.3%)	20 (4.9%)	
	My weight is normal.	132 (58.4%)	179 (43.4%)	

## Discussion

It is imperative to understand the public awareness of obesity and weight management to develop effective health interventions, especially in regions where obesity rates are high [10]. This study focused on the awareness levels among adults in the Asir region, Saudi Arabia, to assess their knowledge and perceptions regarding obesity and weight loss management. This study found that 64.6% of the respondents have a good level of knowledge regarding obesity and weight loss. Similarly, recent research among adults in the western region of Saudi Arabia reported a comparable level of understanding, with 71.8% of participants demonstrating good knowledge [11]. In our study, healthcare professionals and medical students scored notably higher than average, indicating stronger awareness in these groups. By contrast, a study by Duante et al. in the Philippines found that 31.7% of participants had poor knowledge of obesity and weight management, reflecting a lower level of understanding compared to our findings [12]. It is worth noting that differences in scoring criteria, sample populations, and methodologies may account for the variation in results across studies.

Our study revealed low adherence to consistent physical activity routines, a large majority of participants (83.5%) reported not following any specific diet, and only 29.8% engaged in regular physical activity. Of those who exercised, nearly half (47%) reported no exercise days per week, and only 4.7% exercised daily. While the awareness of obesity and weight management is relatively high, practical adherence to healthy habits remains low, pointing to potential gaps between knowledge and behavior. A similar study conducted among Saudi residents revealed a comparable trend, showing that while individuals demonstrated a good understanding of the importance of physical activity in managing obesity, only a small percentage (38.65%) were actively committed to weight loss efforts [13].

Our study further revealed that 43.4% of respondents recognize the relationship between obesity and diet, while 45% believe that high levels of stress can contribute to obesity, identifying stress as a significant risk factor. These findings indicate that although there is some awareness of lifestyle-related causes of obesity, gaps remain in understanding the full range of contributing factors. A study by Acosta et al. supports this observation, reporting that 49.1% of respondents identified age, high stress levels, and consumption of greasy foods as key risk factors for obesity [14].

This study found that a significant proportion of respondents (92.9%) believe that anti-obesity medications, such as Saxenda and Victoza, are unsafe and less effective for managing obesity compared to following a healthy diet and exercising. In addition, most respondents did not prefer using medications for weight loss. By contrast, 1.7% of the respondents held positive views toward weight loss surgery, having previously undergone the procedure. These findings align with prior research by Lean et al., which reported that the majority of the participants (88.6%) preferred weight loss through exercise and diet over surgical methods. While Lean et al. suggested that certain weight loss medications could be viable options, they also acknowledged safety concerns [15]. Moreover, the potential for adverse effects from long-term medication use is notable. For instance, the U.S. FDA ultimately banned the weight management drug lorcaserin due to its association with an increased cancer risk [16].

The results indicate a statistically significant relationship between age, self-perception of weight, and respondents' knowledge of obesity and weight loss management (p < 0.001). Specifically, younger participants in the 21-30 and 18-20 age groups demonstrated higher levels of knowledge (43.9% and 32%, respectively) compared to other age groups probably because the younger generation has increased computer literacy and engagement with technology, which potentially provides greater access to information on health topics. Gender also displayed a marginally significant association with knowledge levels (p < 0.05), with female participants achieving a higher knowledge score (73.5%) than males (26.5%). BMI was similarly associated with knowledge levels with the normal range (18.5-25) exhibiting greater knowledge levels (48.3%, p = 0.008). In addition, individuals who perceived themselves as overweight were more likely to report good knowledge (44.7%, p < 0.001) compared to those who considered their weight normal (43.4%). By contrast, height was not significantly associated with knowledge about obesity and weight loss management (p > 0.05), aligning with previous research findings that also found no correlation between height and knowledge in medical contexts [17].

The study has some considerable limitations. Since it was an online cross-sectional study, it could not determine cause-and-effect relationships between variables, and at the same time, it could not change variables over time. In addition, the online format may introduce selection bias, as individuals without Internet access or those less inclined to engage online were likely excluded, potentially limiting the generalizability of the results.

## Conclusions

The study revealed a moderate level of awareness of obesity and weight management among adults in the Asir region. Despite reasonable awareness, practical adherence to healthy behaviors, such as regular physical activity and dietary management, remains low, indicating a gap between knowledge and lifestyle practices. Most participants recognize lifestyle-related factors contributing to obesity and favor non-surgical, non-medication-based weight management. The study found that variables such as body mass index, gender, age, self-perception of weight, and individual attitudes toward personal weight are associated with a greater understanding of obesity and weight management practices. These factors could therefore be strategically leveraged to target populations with lower awareness and to reduce societal stigma around obesity. 
